# Dr. Claude Clarke Claremont

**Published:** 1911-05-20

**Authors:** 


On April 30 Dr. Claude Clarke Claremont died at Southsea, aged fifty-six. He studied at University College Hospital and at London University, where he graduated M.B., B.S. in 1880 and M.D. in 1886. Previous to this he had qualified as M.R.C.S. in 1877 and L.S.A. in 1878. He had been house-surgeon to the public hospital and dispensary, Sheffield, house-physician to the Seamen's Hospital, Greenwich, and physician to the Royal Portsmouth Hospital, and to the Portsmouth and South Hants Eye and Ear Infirmary. He was a Fellow of the Royal Society of Medicine.

